# Semantic similarity analysis in speech samples of patients with first-episode bipolar disorder

**DOI:** 10.1192/j.eurpsy.2025.582

**Published:** 2025-08-26

**Authors:** B. Arslan, E. Kizilay, E. Bora

**Affiliations:** 1 Department of Neurosciences; 2 Department of Psychiatry, Dokuz Eylul University, Izmir, Türkiye; 3 Department of Psychiatry, University of Melbourne and Melbourne Health, Melbourne, Australia

## Abstract

**Introduction:**

Individuals with first-episode bipolar disorder (FEBD) may have language abnormalities and sub-threshold formal thought disorder (FTD). Natural language processing (NLP) methods that have been shown to assess FTD in psychosis can be used in the early stages of bipolar disorder (BD).

**Objectives:**

The purpose of this study was to examine the differences between FEBD and healthy controls (HC) utilizing NLP methods.

**Methods:**

Speech samples were collected from 20 FEBD and 20 HC while describing eight Thematic Apperception Test images. The manually transcribed text was then processed using word2vec to generate vectors. The semantic similarity between words was computed utilizing a moving window method to windows ranging in size from 5 to 10. Finally, the average, variance, maximum, and minimum of these similarities were calculated.

**Results:**

All mean similarities (windows of 5 to 10) were significantly higher in FEBD (p< .001, p=0.001, p=0.002, p=0.002, p=0.003, and p=0.004, respectively). Additionally, all variances of similarities were highly significant and were increased in FEBD (all p values< .001). Regarding maximum values, except for the window of 5, all of the remaining windows were significantly higher in FEBD (all p values <.05.).

**Conclusions:**

Our findings indicate that semantic similarity increased in the FEBD group compared to HC. Overall, NLP methods offer an easily applicable approach for assessing FTD in FEBD and discriminating between FEBD and HC.

**Disclosure of Interest:**

None Declared

